# Tetrahydroisoquinoline derivatives: a new perspective on monoaminergic dysfunction in children with ADHD?

**DOI:** 10.1186/1744-9081-3-64

**Published:** 2007-12-10

**Authors:** Veit Roessner, Susanne Walitza, Franz Riederer, Regina Hünnerkopf, Aribert Rothenberger, Manfred Gerlach, Andreas Moser

**Affiliations:** 1 Department of Child and Adolescent Psychiatry, University of Goettingen, Goettingen, Germany; 2 Department of Child and Adolescent Psychiatry, University of Wuerzburg, Wuerzburg, Germany; 3 Department of Clinical Neurology, University of Vienna, Vienna, Austria; 4 Department of Neurology, University of Lubeck, Lubeck, Germany

## Abstract

**Background:**

The dopamine-derived tetrahydroisoquinolines (TIQ) synthesized endogeneously from aldehydes and catecholamines have shown to modulate neurotransmission, central metabolism and motor activity. Converging evidence has implicated abnormalities of the dopamine metabolism to the pathophysiology of Attention-Deficit/Hyperactivity Disorder (ADHD). Therefore, four TIQ derivatives involved in central dopamine metabolism (salsolinol, N-methyl-salsolinol, norsalsolinol, N-methyl-norsalsolinol) have been analyzed for the first time in children and adolescents with ADHD and healthy controls.

**Methods:**

42 children and adolescents with ADHD and 24 controls from three sites participated in this pilot study. Free and bound amounts of salsolinol, N-methyl-salsolinol, norsalsolinol, N-methyl-norsalsolinol have been analyzed in urine.

**Results:**

In the ADHD group, free and total amounts of the four TIQ derivatives in urine were significantly higher compared to urine levels of healthy controls. For N-methyl-salsolinol_free_, most of the ADHD patients were identified correctly with a sensitivity of 92.5% (specificity 94.4%).

**Conclusion:**

Urine levels of salsolinol, N-methyl-salsolinol, norsalsolinol and N-methyl-norsalsolinol are elevated in children and adolescents with ADHD and point to a new perspective on catecholaminergic dysfunction in ADHD. However, replication and extension of this pilot study would progress this innovative and promising field.

## Background

Attention-Deficit/Hyperactivity Disorder (ADHD) is a common worldwide disorder characterized by inattention, impulsivity, and hyperactivity. Despite a large amount of research its etiology still remains unclear. Actually, ADHD is regarded as a multifactorial disorder caused by many interacting and/or additive risk factors [1]. There is equivocal evidence from genetic, imaging and medication studies in humans as well as in animal models of ADHD that dopamine and noradrenaline metabolism are affected [1,2]. Current models of ADHD propose a hypofunctioning of e.g. three interacting dopamine systems [3]: (1) the mesolimbic dopamine system primarily associated with altered reinforcement of novel behavior and deficient extinction of previously reinforced behavior, (2) the mesocortical dopamine system associated with deficient attention and poor behavioral organization and (3) the nigrostriatal dopamine system impairing motor functions and causing poor nondeclarative habit learning. But the detailed mechanisms underlying these metabolic impairments are still unknown [4]. Previous studies in ADHD found only a limited relationship of plasma and urine levels of dopamine metabolites to the activity of central dopamine metabolism as well as small effects of stimulant medication on urinary dopamine metabolites [5]. Accordingly, studies on the levels of dopamine metabolites in the cerebrospinal fluid have been performed, but yielded also mixed results of limited value [6-9].

In this context the dopamine-derived tetrahydroisoquinolines (TIQ) including salsolinol and norsalsolinol derivatives are of high interest [10] because of their role as an acute modulator of dopamine and noradrenaline neurotransmission (see [11] for a review). TIQ affect receptor status, enzyme activity of the catecholamine biosynthesis as well as mitochondrial metabolism. Furthermore, exogenously administered TIQ are known to produce changes of motor activity in rodents [12-14].

**Figure 1:**
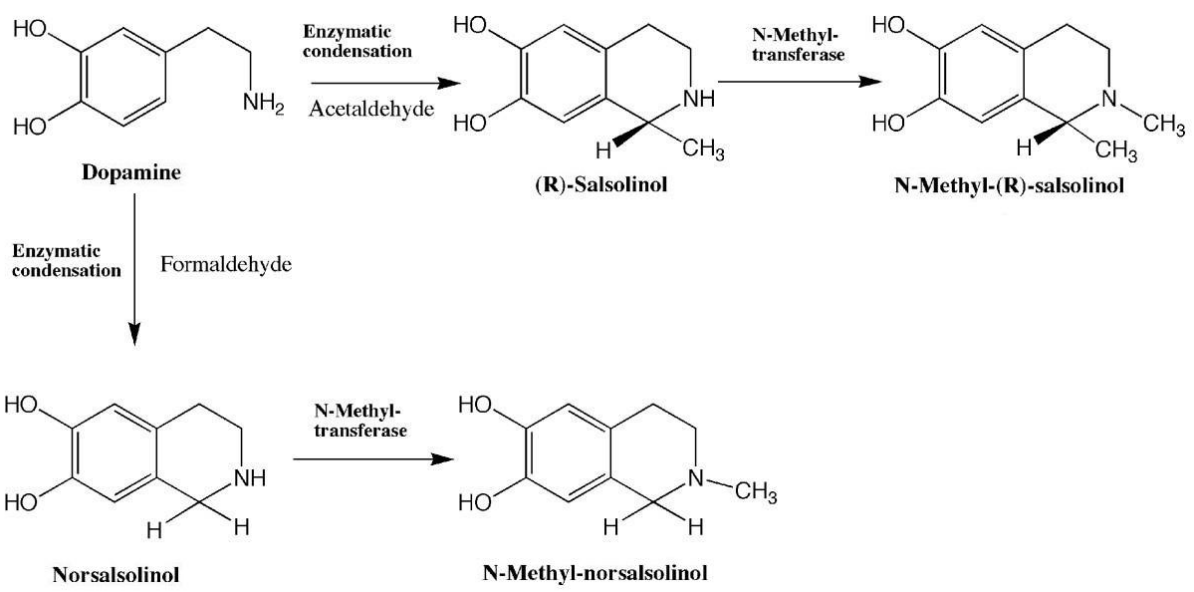
Physiological metabolism of TIQ derivatives.

TIQ are found at low concentrations in postmortem brain [15], cerebrospinal fluid [16] and urine [17] of adults without any neuropsychiatric disorder. In human brain the highest concentration of the TIQ derivative salsolinol and its metabolites have been detected in the basal ganglia [18] – an area implicated in the etiology of ADHD [1]. Thus in ADHD deviations of TIQ levels might indicate disturbances of dopamine and noradrenaline metabolism. In the human brain, two TIQ derivatives salsolinol and norsalsolinol are suggested to be synthesized from dopamine by both a non-enzymatic formation via a Pictet-Spengler reaction and an enzymatic synthesis via a salsolinol synthase [19]; their N-methyl derivatives were formed subsequently enzymatically by N-methyltransferase [20] (Fig. 1).

Because TIQ occur physiologically not only from their in vivo formation but also from ingestion of various foods [21,22], they seem to be worth to be investigated also in the light of the ongoing debate concerning nutritional influences on ADHD symptomatology [23,24].

Because there are still different hypotheses on hyper- and hypodopamine deviances in central metabolism in ADHD [25] and TIQ have never been examined in ADHD, there is no directed hypothesis in our pilot study, i.e. it is unclear if the four TIQ derivatives under investigation are normal or enhanced versus reduced in the urine of children and adolescents with ADHD compared to healthy controls. Thus, the study also serves to generate a hypothesis for further testing in larger samples.

## Methods

### Subjects

42 children and adolescents with ADHD (mean age 12.1, SD 3.2 years) and 24 healthy controls (mean age 23.8, SD 17.0 years) from three sites (Departments of Child and Adolescent Psychiatry of the Universities of Goettingen and Wuerzburg, Department of Clinical Neurology of the University of Vienna) were enrolled. All patients were referred and fulfilled DSMIV-TR [26] criteria for ADHD. 18 patients were on stimulant medication at the day of urine sampling. 16 patients suffered from one or more co-existing psychiatric problems such as conduct disorder (n = 13), learning disorders (n = 4), tic disorders (n = 2) and others (n = 5).

Controls were recruited from hospital staff, their children and a school class near Goettingen. All controls were screened by an expert child and adolescent psychiatrist for absence of psychiatric disorders and reported no medication before and during study participation.

Urine was collected over 12 hours starting at 7 p.m.. Study participants were not allowed to consume food or beverages rich in TIQ derivatives (cheese, chocolate, fresh and dried banana, soya sauce, beer, Port and white wine) for 48 hours before urine samples were obtained [22].

The pilot study was approved by the ethics committees of each participating sites. Informed consent was obtained from the children and their parents. This study was conducted in accordance with the Helsinki Declaration.

### Urine analyses

The 12 h urine samples were collected in the presence of 50 mg semicarbazide and 50 mg Na_2_EDTA. All aliquot samples collected were stored at -40°C and subsequently measured by a two-step chromatography. Urine samples were analyzed at least three times for all conditions, firstly processed by affinity chromatography, and then TIQ derivatives were quantified by high performance liquid chromatography with electrochemical detection (HPLC/ECD) as previously described [17,27]. Under our experimental conditions, free and total concentrations of TIQ compounds could be measured. As described earlier [28], dihydroxylated TIQ derivatives are in part bound to sulfo- or gluco-residues which can be deconjugated by incubation with arylsulfatases and β-glucuronidases. Since conjugated derivatives can not be detected directly, in our study individual bound amounts were calculated by TIQ_bound _= TIQ_total_-TIQ_free_.

### Statistics

Statistical evaluations were conducted using the Statistical Package for the Social Sciences (SPSS, version 12.0). Values were expressed in nM ± SEM as indicated [29]. We performed analyses of variance (ANOVA) to compare the concentrations of the TIQ derivatives among the groups followed by analyses of covariance (ANCOVA) with age, medication (yes/no), and co-existing psychiatric problems (yes/no) as covariates to control for these possible confounders.

## Results

In the ADHD group (n = 42), free and total concentrations of all measured TIQ derivatives were increased in urine samples compared to those of healthy controls (n = 24) (ANOVA, Table 1). In contrast, of the conjugated TIQ forms only the concentration of norsalsolinol_bound _was significantly different between the groups.

**Table 1:** Urine levels of tetrahydroisoquinoline (TIQ) derivatives of children and adolescents with ADHD and healthy controls in nmol.

TIQ Derivative	ADHD		controls		ANOVA	ANCOVA^a)^
	N	concentration mean (SEM)	N	concentration mean (SEM)	F	F
Salsolinol						
*Free*	36	8.44 (2.04)	24	0.06 (0.1)	11.17***	5.87**
*Total*	38	19.34 (5.0)	24	2.79 (1.9)	6.57*	2.82^ns^
*Bound*_*cal*_	23	20.1 (7.2)	3	22.3 (11.0)	0.01^ns^	0.01^ns^

N-methyl-Salsolinol						
*Free*	39	71.13 (8.6)	19	14.54 (4.0)	19.76***	10.31**
*Total*	39	129.01 (17.8)	21	53.51 (6.9)	9.22**	3.12^ns^
*Bound*_*cal*_	37	59.0 (16.2)	19	32.0 (6.7)	1.61^ns^	0.23^ns^

Norsalsolinol						
*Free*	41	553.30 (68.0)	24	121.50 (21.7)	22.64***	25.73***
*Total*	42	1107.89 (123.7)	22	253.09 (53.37)	23.63***	29.84***
*Bound*_*cal*_	39	599.4 (68.3)	17	195.1 (53.9)	13.58***	20.78***

N-methyl-Norsalsolinol						
*Free*	42	9.06 (1.6)	24	0.94 (0.7)	77.07***	12.28***
*Total*	42	33.76 (7.1)	24	10.64 (4.3)	5.38*	6.56*
*Bound*_*cal*_	38	27.3 (6.5)	13	17.9 (6.0)	0.63^ns^	1.88^ns^

a) covariates: age, medication (yes/no), co-existing psychiatric problems (yes/no); no effect of covariates except 'medication status' (*F *= 11.53; *df *= 1; *p *< .001) and 'co-existing psychiatric problems' (*F *= 7.80; *df *= 1; *p *< .01) for N-methyl-salsolinol_free_

* p < 0.5; ** p < 0.01;*** p < 0.001; ns = not significant

Since the ADHD group and the healthy controls differed in age (F = 18.99, df = 1,64, p < .001), and since the control group itself was heterogeneous in age (children: n = 13, mean age 10.5 SD 3.9 years; adults: n = 11, mean age 36.7 SD 11.3 years; F = 62.01, df = 1,22, p < .001), we subsequently performed an ANCOVA with the covariate 'age'. Additionally, because of differences in the ADHD group in medication status (24 without versus 18 with) and co-existing psychiatric problems (26 without versus 16 with) two further covariates were included. There were significant effects of the covariates 'medication status' (F = 11.53, df = 1, p < .001) and 'additional psychiatric diagnoses' (F = 7.80, df = 1, p < .01) only for N-methyl-salsolinol_free_. However, the same results as obtained using ANOVA were found for all measured TIQ_free _derivatives (ANCOVA, Table 1). In addition to the TIQ_free _derivatives, also the concentrations of norsalsolinol_total_, norsalsolinol_bound _and N-methyl-norsalsolinol_total _remained increased in ADHD.

To determine the predictive quality (ADHD yes/no) of elevated TIQ levels (elevated yes/no) sensitivity and specificity were calculated. For the definition of an elevated TIQ level an arbitrary limit was set by the mean of the free concentration of the control group for each TIQ derivative plus one SEM. Specificity is the proportion of true negatives (no diagnosis of ADHD and no elevated TIQ level) of all negative cases (no diagnosis of ADHD = all controls) in the population; sensitivity is the proportion of true positives (diagnosis of ADHD and elevated TIQ level) of all positive cases (all ADHD patients) in the population (sensitivity/specificity (%): N-methyl-salsolinol_free_: 92.5/94.4; norsalsolinol_free_: 87.8/80.0; N-methyl-norsalsolinol_free_: 69.0/93.5; salsolinol_free_: 55.5/95.2).

To exclude possibly confounding effects of age, medication and comorbidity not only statistically by including covariates but also by strict in- and exclusion criteria of both groups, we analyzed in a second step the data of 21 children and adolescents with ADHD compared to 19 healthy controls under the age of 18 years. There were no differences in age (ADHD: n = 21, mean age 11.8 SD 3.5 years; controls: n = 12, mean age 9.5 SD 3.2 years; F = 3.50, df = 1,32, p > .05) and in the ADHD group there were no patients with medication and comorbidity. The group differences in TIQ levels remained as calculated by the ANOVA in the whole sample for salsolinol_free _(ADHD: n = 16, mean = 5.64 SEM 2.02; controls: n = 12, mean = 0.11 SEM = 0.11; F = 5.56, df = 1,26; p < 0.05), salsolinol_total _(ADHD: n = 19, mean = 10.81 SEM 2.17 nmol; controls: n = 12, mean = 0.22 SEM = 0.22 nmol; F = 14.78, df = 1,29; p < .001), N-methyl-salsolinol_free _(ADHD: n = 18, mean = 66.28 SEM 8.27 nmol; controls: n = 10, mean = 10.50 SEM = 1.71 nmol; F = 24.54, df = 1,26; p < 0.001), N-methyl-salsolinol_total _(ADHD: n = 20, mean = 116.52 SEM 13.03 nmol; controls: n = 11, mean = 46.09 SEM = 9.11 nmol; F = 13.85, df = 1,29; p < 0.001), norsalsolinol_free _(ADHD: n = 21, mean = 688.73 SEM 122.37 nmol; controls: n = 12, mean = 66.26 SEM = 14.64 nmol; F = 14.52, df = 1,31; p < 0.001), norsalsolinol_total _(ADHD: n = 21, mean = 1416.67 SEM 211.09 nmol; controls: n = 11, mean = 150.89 SEM = 28.09 nmol; F = 18.45, df = 1,30; p < 0.001), norsalsolinol_bound _(ADHD: n = 21, mean = 727.93 SEM 102.37 nmol; controls: n = 10, mean = 94.70 SEM = 21.54 nmol; F = 17.72, df = 1,29; p < 0.001) and N-Methyl-norsalsolinol_free _(ADHD: n = 21, mean = 11.93 SEM 2.90 nmol; controls: n = 12, mean = 0.44 SEM = 0.26 nmol; F = 8.79, df = 1,31; p < 0.01). Analogously, the absence of group differences remained for N-methyl-salsolinol_bound _(ADHD: n = 18, mean = 52.88 SEM 9.02 nmol; controls: n = 11, mean = 36.55 SEM = 9.31 nmol; F = 1.43, df = 1,27; p = 0.243), N-Methyl-norsalsolinol_bound _(ADHD: n = 18, mean = 30.52 SEM 13.46 nmol; controls: n = 8, mean = 4.75 SEM = 1.68 nmol; F = 1.37, df = 1,24; p = .25). For salsolinol_bound _(ADHD: n = 10, mean = 8.54 SEM 2.41 nmol; controls: n = 1, mean = 2.67) no group comparison could be performed because in the control group only for one child its concentration could be calculated successfully. For N-Methyl-norsalsolinol_total _the significant difference between both groups including all patients changed to a trend (ADHD: n = 21, mean = 38.10 SEM 13.88 nmol; controls: n = 12, mean = 4.81 SEM = 1.51 nmol; F = 3.23, df = 1,31; p = .08).

## Discussion

Comparisons of urine concentrations of TIQ derivatives between children and adolescents with ADHD and healthy controls revealed higher concentrations of salsolinol_free_, N-methyl-salsolinol_free_, norsalsolinol_free_, and N-methyl-norsalsolinol_free _in ADHD patients even when considering three covariates (age, medication status, co-existing psychiatric problems) or when subjects with these confounders were excluded. N-methyl-salsolinol_free _showed the highest sensitivity (92.5%) and specificity (94.4%) of the four TIQ derivatives.

Although Moser et al [30] demonstrated a correlation between salsolinol levels in urine and CSF and a time course study of N-methyl-norsalsolinol in CSF indicated a parallel decline of TIQ derivatives on both sides of the blood-brain barrier [17,31] the evidence for a reliable correlation between TIQ levels in urine and the central nervous system remains limited. However, analyses of urine levels seem to be methodologically and ethically justified as the first step to investigate TIQ in children with ADHD, because in studies analyzing other dopamine metabolites in the CSF, the lack of a control group [9] due to ethical considerations [32] limited the significance of the findings and they were not superior to the results of plasma or urine analyses. Nevertheless, increased central dopamine concentrations might cause increased concentrations of salsolinol and norsalsolinol in urine resulting also in increased urine concentrations of N-methyl-salsolinol and N-methyl-norsalsolinol. This would support the "hyperdopamine hypothesis" of ADHD which is in contrast to the majority of findings indicating hypodopamine neurotransmission in ADHD [3,5] although some authors combined both hypotheses to a comprehensive and more complex model [25].

Interestingly, in the present study there is no hint for an effect of the therapy with the psychostimulant methylphenidate on TIQ levels, although the increase of the endogenously produced synaptic dopamine concentration through inhibition of the dopamine transporter (DAT), which takes up the dopamine into the presynaptic neurons [33], might have led to higher concentrations of salsolinol_free _and norsalsolinol_free_.

In any case, concluding an exclusive relationship between increased central dopamine metabolism and elevated urine concentrations of TIQ derivatives might be an oversimplified view because the found elevation of TIQ levels in ADHD could result not only from primary central but also from peripheral synthesis [17].

Because ingestion of TIQ influences their levels in urine [21,22], participants of our study were not allowed to consume food or beverages rich in TIQ derivatives for 48 hours before urine samples were obtained [22] and so variations of exogenous TIQ or precursor intake might play a minor role for the group differences found. Moreover, the elevated levels of the non-conjugated TIQ_free _derivatives in urine give evidence for endogenous synthesis rather than oral ingestion, because ingested TIQ_free _derivatives will be rapidly inactivated by gluco- or sulfo-conjugation preventing elevated levels of non-conjugated TIQ_free _derivatives in urine [28]. In addition, oral TIQ ingestion seems unlikely to lead to a simultaneous increase of all four TIQ derivatives in the ADHD group since on the contrary exogenous origin would lead to individual amount profiles of the different TIQ derivatives. Nevertheless, nutritional influences on ADHD symptomatology [23,24] can not be completely ruled out because there may be an ADHD specific profile of changes in TIQ metabolism for each TIQ derivative as an addition of the different TIQ sources.

Independently of considerations of causality, the sensitivity and specificity of TIQ urine levels, especially that for N-methyl-salsolinol, which is much better than that of other neurobiological procedures [1] needs confirmation and differentiation by studies including the analyses of other dopamine metabolites as well as including patients with other disorders of dopamine dysfunction (e.g. tic disorders, schizophrenia). Additionally, a further study with larger sample size should differentiate between ADHD subtypes. Since hyperactivity was found after injection of TIQ in rodents [12-14], in ADHD there might be the strongest correlation between the core symptom hyperactivity and TIQ derivatives as well as the highest urine levels in the predominantly hyperactive-impulsive subtype.

## Conclusion

In conclusion, urine levels of salsolinol, N-methyl-salsolinol, norsalsolinol and N-methyl-norsalsolinol are elevated in children and adolescents with ADHD and point to a new perspective on catecholaminergic dysfunction in ADHD. Replication of the findings in a larger sample of children and adolescents with ADHD focusing on subtypes and including a control group of children with other movement related catecholaminergic disorders would progress this innovative and promising field of research.

## Abbreviations

Attention-Deficit/Hyperactivity Disorder (ADHD), Tetrahydroisoquinolines (TIQ), analyses of variance (ANOVA), analyses of covariance (ANCOVA).

## Competing interests

The author(s) declare that they have no competing interests.

## Authors' contributions

*VR *contributed to the conception and design of the study and was primarily responsible for the interpretation of the data and writing of the manuscript. *SW *contributed to the study design and data acquisition. *FR *contributed to the study design and data acquisition. *RH *contributed to the study design and data acquisition. *AR *contributed to the design of the study and revision of the final manuscript. *MG *contributed to the conception and design of the study and the interpretation of the data and writing of the manuscript. *AM *contributed to the conception and design of the study, to the analyses and the interpretation of the data and writing of the manuscript. All authors have read and accepted the final manuscript.
